# Effect of Constitutional Disease on the Health and Structure of the Human Teeth

**Published:** 1839-09

**Authors:** James D. McCabe

**Affiliations:** Surgeon Dentist, of Richmond Va.


### EFFECT

### *Of Constitutional Disease on the Health and Structure of the Human Teeth,*

[ BY JAMES D. McCABE, SURGEON DENTIST, OF RICHMOND VA. ]

Numerous evidences exist to convince us that the human teeth attracted attention at a very early period of time; as far back as the records of man extend, they are spoken of as essential attributes to a lovely countenance. The ancient poets and inspired writers unite alike to celebrate their utility and beauty, enumerating amongst the charms of female loveliness, the whiteness and regularity of the teeth. But while they speak of their appearance, and evidence their regard for their beauty and regularity, we have no authority for supposing that the diseases which *now* afflict those organs, *then* existed. The world was yet in the morning of its youth, and the grievous ills incident to the violation of natural *organic law,* were but partially developed.

To the observant mind, nothing can be more distinctly obvious, than the prominent truth, that the human constitution, weakened by excess and debauchery, constituted the grand cause of the ills which afflict humanity, and scatter death through the abodes of man, and are more destructive to his happiness, than the clouds of Locusts, to the green and fruitful fields of Egypt. The poet wisely, truly sung,—

"The first Physicians by debauch were made,
"Excess begun, and *still* prolongs the trade."

The teeth, like other organized members of the human system, are subject to be affected by the same causes which operate on the human system generally, or locally. In other words, the diseases are mainly produced by departures from natural law; which, affecting the general constitution, involved in their effects all parts of the system, weakening and disordering their functional operations, thereby affecting the formative process; these results are by a law of hereditary transmissions entailed upon our offspring, and thus diseases become general or local, according as our habits tend to their developement in the one form or the other. I am aware that in the remarks I have to offer, I may differ from my professional brethren—this I regret—and as an enquirer after truth, am ready to submit to correction if in error; but under present advices, I

feel called on to maintain views founded, not on theories of the study, but upon observations, and in coincidence with philosophical principles. If it be true, as all experience hath shewn, that self indulgence and luxury have ever a tendency to enervate the constitution, and produce disease—and, that as a people indulge in these habits, so in equal ratio, the forms of old diseases are changed in intensity, and new ones introduced; then it must follow, as a necessary consequence, that if the teeth compose *any part* of the system thus affected, they have, and still do participate in all these changes, not only in their general health, but in form and structure. I have been at a loss to understand the object of those, who have spoken of the teeth, as though they were independent members of the human system, separated entirely from all connection with the law of sympathies; frequently inflicting great injury by nervous transmission, yet unaffected in return. These writers have contended that climate and habits could exert no influence over these organs, as long as external agents were excluded from contact with them. These *notions* have been the mischievous cause of the *Quackery* with which the dental profession has been cursed; nor have the medical practitioners of our country been wholy blameless in this matter;—they have taught that "a little mechanical skill in the use of the small tools," was the perfection of dental qualifications, and thus from *Maine* to the Gulph of Mexico, the land has been inundated with ignorant pretenders, until the profession is so much degraded, that in some neighborhoods, a DENTIST *is considered Cousin German to a dealer in wooden nutmegs.* It is perfectly reasonable that thousands should adopt the dental profession, when it was of such easy access as represented. Who could not acquire, in a few weeks, a little manual dexterity in the use of the key or forceps, and learn to fill up a decayed tooth with tin foil, or an amalgam of quicksilver and bismuth? Nothing easier! But this is a flagrant departure from our subject, for which the reader must excuse us. The position is, that diseases of the teeth may be traced to the effects produced by departure from organic law, whereby the original healthy constitution was impaired, and the physical structure subjected to disease;—and, that at this day, they are more or less affected according to the habits and general health of any people.

The earliest allusions made to the teeth, are to be found in the sacred writings; and notwithstanding these allusions are frequently referred to in proof of the antiquity of the dental profession, not a solitary "loop whereon to hang" such a conclusion is furnished by them. That they were admired, and spoken of with sentiments of admiration, is true; but not one word is said of their diseases, which would no doubt have been done, if then, as now, they were extensively diseased. A late writer, to whom the profession is much indebted for a mass of practical information, and deep devotion to its interests, has fallen into this error. He remarks, speaking of the teeth, and sustaining the theory we object to, that, "The Hebrews considered the loss of these organs as a grievous, and somewhat disgraceful circumstance," and in evidence of this, quotes David's prayer against the wicked judges: "Break out their teeth, O God, out of their mouth!" But David prays in another place, that the enemies of God may be "smitten on the cheek bone." Are we then to infer that they were subject to diseases of this class also, and that they considered the loss of the cheek bone "a grievous and somewhat disgraceful circumstance ?" The fact is, that any mutilation of "the human form divine," was by them considered "grievous," and "disgraceful," and the quotation no more proves the existence of disease in the one case, than in the other. Disease of any form was so unusual among this people, until they abandoned their frugal and temperate course of life, that they were regarded as special emmissaries of the Most High, sent to punish individual sins; or as a scourge to nations. Indeed, the history of every nation will amply confirm the assertion, that as they continued in habits of simple frugality, they were free from diseases; a departure from these habits generated their production, and thus from the operation of a natural law, "the sins of the fathers have been visited upon the children" from generation to generation. The first historian who gives us any clue to ancient disease, is HERODOTUS; he speaks of the subdivisions of the healing art in Egypt, and enumerates in the list those who attended to the teeth. Egypt was, in civilization and refinement, the oldest country on the face of the globe; the habits of its people were luxurious and enervating; and it is more than probable, that remittant and intermittent fevers prevailed to a considerable extent; the inundations of the Nile would at least present a fruitful cause of production to this disease. The healing art, was of consequence, very early cultivated amongst that people, and as the various branches of practice were given to different persons, what more natural than the treatment of diseased teeth, which according to the foregoing shewing, must have participated in the morbid and acute affections to which the system was subjected, illustrating the operation of that general law of our nature, which sends through the human system the poisonous stream of disease, more or less affecting every part.

Hippocrates subsequently, in his descriptions of the teeth, most evidently shews that he believed the health of these organs dependant on the general health of the system, and that the teeth are so intimately connected by sympathy, with other parts, that the local irritation of dentition, will produce constitutional disease, and *vice versa* constitutional irritation, will produce local diseases of the nerves and membranes of the teeth. It is said that the teeth are at this day generally diseased in the city of Rome. Now there is no clearer evidence of the effects of constitutional disease on the character of the teeth to be found. The ancient city of Rome, and its environs, in the day of its iron power, when its citizens were frugal and temperate, were considered the seat of health; but as it has become enervated by excess, and neglect of good habits, its diseases have multiplied to an alarming degree, until its *Pontine marshes* are said to pour forth their pestiferous malaria of death, for the annual destruction of thousands; hence the increase and existence of dental diseases. A host of testimony of this description could be adduced to shew, that as the habits of any nation have contributed to a vigorous constitution, the teeth have been generally good; and as the habits have been enervating and luxurious, contrary effects have been produced. I wish here to remark that I do not deny the bad effects produced by external agents and merely accidental causes. My attention has been specially directed to this subject by a comparison between the teeth of those living in the high mountainous country of Virginia and North Carolina, and those residing in the lower portions of those States. In the former regions of country, the inhabitants are generally frugal and temperate in their habits, possessed of vigorous constitutions, and breathing an invigorating and salubrious air. Fevers, in their various forms are of rare occurrence, and the teeth well formed and seldom subject to disease, unless produced by external causes. In the latter country, the people are wealthy, luxurious in their habits, hospitable in their disposition. They are surrounded by extensive swamps from which noxious exhalations, and deadly miasmata are continually arising, polluting the atmosphere, and destroying health. The *Dæmon Fever* shakes an autumnal pestilence from his wings, and converts a land resembling the aluvial Delta of the Nile, smiling in the richness of its production, and exuberance of its vegetable life, into an extensive golgotha—a vast charnal-house of death. The difference in the general health, is not greater than the marked distinction which exists, in the diseases and organization of the teeth. Making due allowance for the exceptions which exist to every general rule, I hesitate not to say, that the teeth are as much affected by these circumstances, as any other organ of the general system. I am aware that it may be urged, that the prevalence of fever necessarily requires the extensive exhibition of mercurial medicines, and that the tendency of this remedy is to vitiate the secretions of the salivary ducts, and produce morbid affections of the glands, and that thus the injury is produced. But this is obviously incorrect. In no one instance have I ever seen or heard of the destruction of the *structure of the teeth* from these causes, though the gums and processes have in many instances been entirely removed by excessive salivation, and not unfrequently adhesions formed between the gums and membranes of the cheek. The effect of fever is to debilitate the system generally, and produce a tendency to functional disorder in all the organs. This debility is transmitted from generation to generation, and is seen as fully operating in the parts engaged in the formation of the teeth, as in any parts of the system, as is evidenced in the delicate organization, and speedy decay of these valuable appendages. I have seen infants' first teeth come through the gum much affected with caries, and in one case, a little boy, who cut his first desideous teeth (the two lower central incisors,) when he was seven years old; I called to see him about a week after they were developed, and found them extensively affected with *denuding of the enamel.* It is a most melancholy sight in many of our towns, in the lower part of the states alluded to above, too see almost every child you meet with, from the first appearance of the temporary teeth, to the full developement of the permanent ones, smiling in infantile playfulness as they disclose a mouthful of blackened crowns, and ulcerated gums; like (as a medical friend remarked, when speaking of these things,) " the blackened stumps of a new ground partly grubbed up." And females too, with faces fair as Mohommed's Houries, and charms that would warm an Anchorite's heart, uttering tones like the song of the *Bulbul* from lips lacerated with the spiculated fangs of decayed teeth. Whence these effects, if the diseases of climate exercise no influence over the health of the teeth? If constitutional debilities are not propagated by transmission to our offspring, and these seeds of disease nurtured and strengthened by our habits? In view of these things, I hesitate not to say, that as temperance and frugality prevail, so will humanity be delivered not only from this, but all the multiform inflictions of disease to which "flesh is heir."—It would, I admit, require centuries to eradicate the diseased taint from our systems; yet it would yield to a well ordered constitutional frugality, and the world be delivered from dentists and doctors, who to effect "a consummation so devoutly to be wished," would, I doubt not, gladly contribute their aid. Of one thing we may be assured; the benevolent author of our being did not intend our teeth to be diseased, more than other parts of the system; and we may, I think, rationally conclude, that if the eye, the ear, the bones, &c., participate in the general and local affections of constitutional disorders, it would be excluding the teeth from the laws of the animal economy to deny their participation. Another striking proof of the doctrine of this paper, is found in the history of diseases of the teeth, amongst the blacks of our lower southern country. The general impression is, that their teeth are rarely affected, and upon this hypothesis, has been predicated the doctrine, that hot and cold solids and fluids, were the great exciting causes of *caries* of the teeth; these opinions are erroneous; the same difference will be found to exist with the blacks, that I have spoken of with regard to the whites. The character of their teeth is determined by habits and general health;—the plantation hand who has his four pounds of meat, and half bushel of meal per week, though constantly exposed to the sun, laboring in the field, day by day through the warmest season of the year, is healthy; and strange as it may appear, has less constitutional tendency to fever than the white man; his teeth are good, and it is very seldom the case that it is necessary to extract them. But on the other hand, the house servant who shares in the master's mode of life, and in his degree becomes enervated by the habits of life he there contracts, and the excessive feeding of the white family, is subject to the same diseases with them, and as a consequence suffers from diseased teeth. I have examined the teeth of field hands, aged from 40 to 50, and though imbeded in salivary calculus, they were perfectly sound, not one having been removed. While examinations have proven that in house servants of the same age it is a rare thing to find a molar tooth remaining; of course the relative health is included in the statement.

I have before remarked, that external agents were to be considered accidental causes of diseased teeth; and so perfectly am I convinced of this fact, that I believe a tooth formed under a healthy and vigorous constitutional direction will resist for a *long time*, the action of many of the essential oils. Indeed, I am informed that Professor Dunglison, while filling a chair in the University of Virginia, recommended to his class, *a weak solution of sulphuric acid and water, as a pleasant and safe dentifrice;* his own experience, and practice for years, being the ground of recommendation. While I dissent from the professor's dentifrice, and would condemn in no measured terms the quack nostrums for the teeth, vended in the form of powders and tinctures, I nevertheless believe that we attach too much importance to external agents in our remedial treatment of the teeth. The fact is, that in all countries, where diseases, especially fevers prevail to any extent, the teeth affected by this cause in their formation, have a tendency to disorganization. The action of external agents only gives a speedy developement to this tendency, but does not constitute the primary cause. It is, therefore necessary that the dental practitioner should not only know *how,* but *when,* to perform an operation, also to understand the remedial treatment necessary to render the operation perfect. If the views we have advanced, be correct, it will be found that much of the abuses heaped upon physicians for destroying the teeth, is unmerited, and that the rapid decay of teeth immediately succeeding a protracted spell of sickness, is more the result of disease extending to these organs, than the effect of medicines exhibited during its prevalence. The dentist should always be prepared, when called in to prescribe for the relief of all such cases, to conduct his operations in view of the causes producing the affection, thus mingling a knowledge of human diseases generally, with the manipulations of his art, under the direction of, and in coincidence with, the medical attendant; for in a vast number of cases, the advantages of an operation are destroyed by the circumstances under which it is performed; and though the operator may possess "a little mechanical skill in the use of small tools," he will find that something more is needed in the honest and conscientious practice of the profession he has chosen. I have seen vast benefits resulting to the teeth, from a mild alterative treatment, directed to the removal of morbid diathesis of some important organ; and again, a local treatment of the teeth, entirely removing constitutional disorder. It requires, therefore, some medical acumen to detect the cause of disease, and prevent an attempt to treat the effects alone. I have no doubt the observations of others have suggested similar thoughts; and as the "Journal" promises much to the profession, I have sent you these desultory remarks, more to elicit enquiry than for any other purpose. If they are worthy of a place on a spare page, you can thus appropriate them. (A)

---

I am so much pleased with the sentiments contained in Mr. Shepherd's article on a preceding page of the present number, that I feel it my duty to say, that notwithstanding forceps have been greatly improved by the efforts of Dr. Flagg, of Boston, and other dentists, distinguished for their skill and ingenuity, and can with the utmost propriety be recommended for general use in the extraction of teeth, yet I have never abandoned the use of the improved key-instrument, and regard it as decidedly the best extracting instrument in my case. In a general way, it can be used in all cases in which the forceps are available; but not always with the same facility or convenience; for there are many cases where the forceps cannot be used successfully without cutting away considerable portions of the socket, and subjecting the patient to much pain and inconvenience during the operation, the whole of which can be avoided by a judicious application of the key; and in all such cases there is no other instrument in use, or that ever has been used to my knowledge, which will answer the same good purpose. From the injuries that are almost daily inflicted by it, and which so frequently come under our observation, I am led to the conclusion that there are but a few of the great mass of "Tooth drawers," and dentists that thoroughly know its use or understand its application. N. Y. ED.

(A) In publishing this communication from the pen of Dr. McCabe, we would not be understood as becoming, in any sense, sponsors for the truth of its doctrines, any more than we do for any other opinions which our correspondents may choose to discuss on the pages of the Journal. We esteem it our duty as Editors and Publishers of a Dental periodical established exclusively for general benefit, to open our columns to free discussion, and on these principles we give place to Dr. McCabe's article with great pleasure.

